# Running Performance of High-Level Soccer Player Positions Induces Significant Muscle Damage and Fatigue Up to 24 h Postgame

**DOI:** 10.3389/fpsyg.2021.708725

**Published:** 2021-09-14

**Authors:** Lucas Albuquerque Freire, Michele Andrade de Brito, Natã Sant’anna Esteves, Márcio Tannure, Maamer Slimani, Hela Znazen, Nicola Luigi Bragazzi, Ciro José Brito, Dany Alexis Sobarzo Soto, Daniel Gonçalves, Bianca Miarka

**Affiliations:** ^1^Department of Fights, Postgraduate Program in Physical Education, School of Physical Education and Sports, Federal University of Rio de Janeiro, Rio de Janeiro, Brazil; ^2^Department of Neuroscience, Rehabilitation, Ophthalmology, Genetics, Maternal and Child Health (DINOGMI), Section of Psychiatry, University of Genoa, Genoa, Italy; ^3^Department of Physical Education and Sport, College of Education, Taif University, Taif, Saudi Arabia; ^4^Laboratory for Industrial and Applied Mathematics, Department of Mathematics and Statistics, York University, Toronto, ON, Canada; ^5^Postgraduate Program in Physical Education, School of Physical Education and Sports, Federal University of Juiz de Fora, Juiz de Fora, Brazil; ^6^Escuela de Kinesiología, Facultad de Salud, Universidad Santo Tomás, Santiago, Chile

**Keywords:** muscle damage, fatigue, muscle strength, sports, task performance and analysis, external loads

## Abstract

This study aimed to determine the impact of a soccer game on the creatine kinase (Ck) response and recovery and the specific Global Positioning System (GPS)-accelerometry-derived performance analysis during matches and comparing playing positions. A sample composed of 118 observations of 24 professional soccer teams of the Brazil League Serie A was recruited and classified according to playing positions, i.e., Left/Right Defenders (*D* = 30, age: 25.2 ± 5.8 years, height: 187 ± 5.5 cm, weight: 80 ± 5.8 kg), Offensive Midfielders (OM = 44, age: 25.1 ± 0.2 years, height: 177 ± 0.3 cm, weight: 73 ± 1.2 kg), Forwards (*F* = 9, age: 25.1 ± 0.2 years, height: 176.9 ± 4.3 cm, weight: 74.5 ± 2.1 kg), Left/Right Wingers (*M* = 23, age: 24.5 ± 0.5 years, height: 175 ± 1.1 cm, weight: 74 ± 4.4 kg), and Strikers (*S* = 12, age: 28 ± 0.2 years, height: 184 ± 1.0 cm, weight: 80 ± 1.4 kg). Blood Ck concentration was measured pre-, immediately post-, and 24 h postgame, and the GPS-accelerometry parameters were assessed during games. Findings demonstrated that Ck concentrations were higher at all postgame moments when compared with pregame, with incomplete recovery markers being identified up to 24 h after the game (range: 402–835 U/L). Moreover, Midfielders (108.6 ± 5.6 m/min) and Forwards (109.1 ± 8.3 m/min) had a higher relative distance vs. other positions (100.9 ± 10.1 m/min). Strikers [8.2 (8.1, 9.05) load/min] and Defenders [8.45 (8, 8.8) load/min] demonstrated lower load/min than Wingers [9.5 (9.2, 9.8) load/min], Midfielders [10.6 (9.9, 11.67) load/min], and Forwards [11 (10.65, 11, 15) load/min]. These results could be used to adopt specific training programs and recovery strategies after match according to the playing positions.

## Introduction

Studying the determinants of performance outcomes and recoveries of professional soccer players, such as sprints, accelerations, decelerations, changes in direction, jump movement patterns, technical skills, and tactical actions associated with high-intensity efforts translated into metrics, could potentially be useful to inform the construction of specific conditioning drills in an evidence-based fashion (Ade et al., 2016). In quest of best performance, soccer athletes, coaches, and physical trainers have to decide how and when they have to invest their energy (Akubat et al., 2018). The performance analysis of soccer matches has been increasingly utilized during the previous years for this purpose (Sarmento et al., 2008; Enes et al., 2021). Due to the difficulties and challenges in conducting physiological measurements during a match, studies interested in the time-motion analysis used running performance [Global Positioning System and Local Positioning System (GPS/LPS)] and factors affecting performance outcomes to infer the metabolic profiles of soccer matches (Anderson et al., 2019; Gantois et al., 2020).

The studies of performance analyses showed that soccer match requires many physically demanding performances. The available scholarly literature computed a total and relative distance covered during the game between ∼8,000 and 10,500 m, with a range of ∼100–120 m/min per match (Reinhardt et al., 2019). The low-intensity running performance has not been found determinant in intra-game comparisons, as shown in the published studies (Di Salvo et al., 2010; Modric et al., 2019). In contrast, besides scoring the goals, accelerations, decelerations, the number of sprints and distance covered greater than 18 km/h, and other running metrics variables seem to be the key factors to succeed in professional soccer matches (Mara et al., 2015; Abbott et al., 2018).

Physical performance during a soccer match is highly variable and depends on many factors, such as match intensity, period of the season, age, and playing positions, among others. Several investigations have studied the physical demands of a soccer match across playing positions. The majority of them categorized positions into defenders, midfielders, and attackers. Felipe et al. (2019) reported that defenders covered greater total distance (10,307.33 ± 1,206.33) when compared with midfielders (7,705.06 ± 3,201.10) and attackers (7,240.61 ± 3,411.31) (Felipe et al., 2019). In contrast, another study showed that defenders had lower total absolute distance and work rates when compared with midfielders in the first half and midfielders and attackers in the second half (Vescovi and Favero, 2014). Regarding moderate- and high-intensity running, contradictory results have been reported in the literature (Hewitt et al., 2014).

More in detail, when specifically categorizing the playing positions, central defenders performed lower total distance covered, high-speed running (HSR), and very-high-speed running (VHSR) compared with full-backs, central midfielders, external midfielders, and forwards. Center-backs reported the lowest values for total distance covered (Mendez-Villanueva et al., 2012) and high-intensity activities (Andrzejewski et al., 2009; Buchheit et al., 2010; Brito et al., 2017; Varley et al., 2017); midfielders and second attackers performed the highest total distance covered; wide midfielders and attackers demonstrated the highest peak game speeds and frequency of high-intensity activities (Buchheit et al., 2010; Al Haddad et al., 2015; Izzo and Varde’i, 2017).

Furthermore, participation in a soccer match can lead to acute and residual fatigue, characterized by a decline in physical performance over the following hours, which can persist even for days (Aquino et al., 2016). The magnitude of these disturbances increases within the first 24 h after a competition with peaks between 24 and 48 h post-match (Aquino et al., 2016). Together with a decrease in running performance, the potential insurgence of muscle damage and the increased levels of intramuscular enzymes, such as creatine kinase (Ck) and inflammatory/immunological biomarkers, are reported following soccer competition (Russell et al., 2016; Oliveira et al., 2019). Some studies have found a significant correlation between running performance outcomes and muscle damage markers (e.g., muscle soreness, Ck) at 24, 48, and 72 h after the soccer matches (Russell et al., 2016; Silva et al., 2018). More in detail, Russell et al. (2016) investigated, from a quantitative standpoint, the associations between GPS/LPS-accelerometry findings (i.e., high-intensity distance covered, HSR distance, and the number of sprints carried out) and changes in Ck levels and peak power output during the execution of countermovement jumps 24 and 48 h after the match in a sample of 15 English Premier League Reserve team players. Statistically significant correlations with coefficients ranging from 0.363 to 0.410 were found 24 h but not 48 h post-match. Silva et al. (2018) performed a systematic review and meta-analysis concerning the match-induced fatigue and related recovery profiles of soccer players, taking into account several parameters (i.e., physiological, neuromuscular, biochemical/endocrinological, perceptual, and technical). The authors pooled together 77 studies and computed 1,196 effect sizes (ESs), finding small-to-large variations in the variables under study. These changes could be detected, differently from the previous study (Russell et al., 2016), until 72 h post-match, indicating a persistence of the muscle damage in terms of biochemical, inflammatory, and immunological parameters. These contrasting findings can be reconciled, assuming that some variables (such as those endocrinological/hormonal and technical) can be fully recovered in the short term after the match; for others, the process and dynamics of homeostatic balance are more complex, requiring more than 72 h.

It is well known that the magnitude of muscle damage and the other physiological alterations elicited by matches are associated with oscillations in Ck levels (Coppalle et al., 2019) and related running performance can be assessed with *ad hoc* performance analytical tools (Milanović et al., 2020; Enes et al., 2021; Strauss et al., 2019). Furthermore, soccer matches can affect the players differently depending on their playing positions (Abbott et al., 2018). However, the influence of this variable has been relatively overlooked in the available scholarly literature. Furthermore, with the positional difference of physical demand during soccer matches (Abbott et al., 2018), information about the effect of a soccer match on muscle damage postgame was lacking. Consequently, a comprehensive assessment in terms of positional differences of the muscle damage post-match of elite soccer players is necessary to inform applied practitioners working with soccer players to: (1) tailor and personalize interventions based on the specific needs of athletes, rather than relying on a “one-size-fits-it-all” approach; (2) better adopt position-specific recovery strategies; (3) appropriate time between match and session training; and (4) reduce the risk of injuries and achieve optimal performance outcomes. We formulated the working hypotheses that: (1) there is a difference in terms of performance outcomes among players of different playing positions; and (2) game load can impact Ck responses after competition until 24 h after the match. Therefore, this study was devised to fill in this gap in knowledge and aimed to determine the impact of a soccer game on the Ck response, recovery, and specific running performance outcomes, stratifying by playing position.

## Materials and Methods

### Sample

From an initial list of 800 soccer matches performances, this study randomly selected the performances of 118 athletes from professional soccer teams of Rio de Janeiro during the 2018 and 2019 championship seasons. During the games, athletes were classified as Left/Right Defenders (*D* = 30, age: 25.2 ± 5.8 years, height: 187 ± 5.5 cm, and weight: 80 ± 5.8 kg), Offensive Midfielders (OM = 44, age: 25.1 ± 0.2 years, height: 177 ± 0.3 cm, and weight: 73 ± 1.2 kg), Forwards (*F* = 9, age: 25.1 ± 0.2 years, height: 176.9 ± 4.3 cm, and weight: 74.5 ± 2.1 kg), Left/Right Wingers (*W* = 23, age: 24.5 ± 0.5 years, height: 175 ± 1.1 cm, and weight: 74 ± 4.4 kg), and Strikers (*S* = 12, age: 28 ± 0.2 years, height: 184 ± 1.0 cm, and weight: 80 ± 1.4 kg). We considered the level and locations of opponents: 39 international games of *Copa Libertadores da América*, 58 national games of *Brasileirão Série A*, and 26 state games of *Campeonato Carioca de Futebol*, with 28 teams in different rounds or championship phases, with competitions occurring once per week. The performance-analysis-related data were collected at single time points while the Ck level was studied before, after, and 24 h after the game. Therefore, a total of 354 samples of Ck concentration of all professional soccer players were analyzed in three moments as follows: first, before the game (Ck *n* = 118); second, after the game (Ck *n* = 118); and third, after 24 h of the game (Ck *n* = 118). The sample size was enough to compute estimates with 95% CI and 5% of margin of error.

Each athlete had a minimum of 6 days of rest from the previous match to prevent stress interference, competed in national and international representative championships once (∼90 min) per week, and had been regularly training 2 h of technical and tactical aspects 4–7 times a week and 1 h of physical preparation 2–3 times a week. Therefore, all participants were from the Brazil league and had previous experience with professional soccer events, rules, and procedures used during the event.

The inclusion criteria were as follows: (1) being from the Brazilian league; (2) having played during ∼85% of the game; (3) having a minimum of 6 days of rest from their previous match to prevent stress interference; (4) having competed in national and international representative championships once (∼90 min) per week; and (5) 2 h of regular training of technical and tactical aspects 4–7 times a week and 1 h of physical preparation 2–3 times a week. We excluded participants who played games for more than 90 min, substituted players, and goalkeepers.

This study was approved by the Local Committee of Ethics in Research (No. 13846919.8.0000.5257), following the rules of resolution of the National Health Council and in accordance with the WMA Declaration of Helsinki. Then, the volunteers (age: >18 years) were contacted by the researchers in such a way to be informed about the aims and procedures of the study and signed an informed consent form to participate in the data collection. Measurements were performed before, after, and 24 h after the game. No modifications were made in the training, nutritional, or hydration status of participants, and they maintained a passive recovery time pattern of 24 h without training efforts between the game, postgame, and 24 h postgame.

### Physical Performance Demands

The subjects wore a GPS unit (Catapult Innovations, Scoresby, Australia) during each trial (Jennings et al., 2010b). The performance analyses of the professional soccer players were monitored using a portable 5-Hz GPS unit (Catapult, Melbourne, Australia) during games. The GPS unit was positioned *via* an elasticized shoulder harness to sit between the scapulae of the player at the base of the cervical spine (Petersen et al., 2009a). The GPS unit was activated and a GPS satellite lock was established for at least 15 min before the player taking the field, as per the recommendations of the manufacturer (Petersen et al., 2009b). The recorded information was downloaded after each session using Catapult Sprint software (Catapult Innovations, Melbourne, Australia) for analysis. Once downloaded, the competition data were edited and split into two 45-min halves (Abbott et al., 2018).

Only subjects completing the entire match were included in the analysis process. The mean number of satellites and the horizontal dilution of position were recorded during the data collection (Abbott et al., 2018). The performance analysis followed a preceding protocol (Abbott et al., 2018). The total distance (m), i.e., distance traveled during all the game; total distance by minute; percentage of distance traveled, low-intensity running and jogging (<14 km/h), running (>14 km/h), and sprinting (>18 km/h), distance and number of sprints (>18 and >24 km/h), maximum speed (km/h), number of accelerations (>9 km/h), and deceleration (<9 km/h), jumps (>30 cm), and efforts (i.e., accelerations, deceleration, and jumps) were the performance analysis factors assessed during professional soccer games with ∼90 min of durations (Abbott et al., 2018).

### Ck Measure

Blood Ck concentration was measured pre-, post-, and 24 h postgame by reflectance photometry at 37°C using the Reflotron Analyzer Plus (Reflotron Plus, Roche, Germany), previously calibrated. To reduce errors, only one evaluator was responsible for these collected data. A lancet device with an automatic trigger was used for puncturing the finger after finger asepsis using 70% ethyl alcohol, and the blood was drained into a strip for specific examination (using heparinized capillary strips). A blood sample (32 μl) was immediately pipetted into a Ck test strip, which was introduced into the instrument. The absolute values of Ck (U/L) were used for analysis, according to the study of Aquino et al. (2016).

### Statistical Analysis

The descriptive data are presented as mean and SD, using the coefficient of variance (CV, %) as the measure of variability. The Kolmogorov–Smirnov test (K-S) was used to determine the normal distribution of the data, considering *p* ≤ 0.05. A repeated measure ANOVA was performed to verify the Ck modifications, and a generalized estimating equation (GEE) mixed-linear model accounting for individual (random) effect was conducted, considering the level and location of the opponent (International competitions in South America × National competitions in Brazil × State competitions in Rio de Janeiro) as a control variable. The ES was calculated using eta-squared and interpreted as follows: small (0.01 < ES < 0.06), medium (0.06 < ES < 0.14), or large (ES > 0.14). The significance level of *p* ≤ 0.05 was used. All analyses were conducted using SPSS for Windows software (version 20.0; SPSS, Inc., Chicago, IL, United States).

## Results

Table 1 shows the descriptive analysis of distance and load during the game, with the total/minute ratio separated by the position of the player.

**TABLE 1:** Descriptive analysis of behavior and performance factors, considering each player’s position.

**Factors/Position group**	**Strikers (*n* = 12) M ± SD**	**CV%**	**Wingers (*n* = 23) M ±SD**	**CV%**	**Midfielders (*n* = 44) M ± SD**	**CV%**	**Forwards (*n* = 9) M ± SD**	**CV%**	**Defenders (*n* = 30) M ± SD**	**CV%**
Total Distance (m)	7359.0 ± 1391.1^*#β^	18.9	9199.8 ± 662.1	7.2	10354.1 ± 757.3	7.3	8155.6 ± 1058.6^*β^	13	7830.6 ± 1227.5^*β^	15.7
Distance (m)/min	94.3 ± 1.2^*ψ^	1.3	99.6 ± 5.8^*ψ^	5.8	108.7 ± 6.1	5.6	103.4 ± 7.4	8	90.4 ± 7.8^*ψ^	8.6
Total Load (index)	654.7 ± 118.7^$^	18	871.7 ± 49.0^ψ^	5.6	1047.1 ± 119.0	11.3	1005.0 ± 48.1	4.8	752.0 ± 106.8^$^	14.1
Load/min (index/min)	8.3 ± 0.58^$^	6.9	9.3 ± 0.52	5.7	11.1 ± 1.3	12	10.5 ± 0.71	6.7	8.8 ± 0.8^$^	9
Speed max (km/h)	29.3 ± 1.8	6.2	31.2 ± 1.8	5.8	29.5 ± 2.0	6.7	29.2 ± 2.1^$^	7.2	28.2 ± 2.4	8.6
Acceleration (frequency)	34.2 ± 7.3^#^	21	37.1 ± 8.6^#ψ^	23	31.3 ± 7.5^#^	24	38.6 ± 7.9	20.4	25 ± 6.6	26
Deceleration (frequency)	33.6 ± 9.4	28	41.7 ± 9.0	22	37.1 ± 9.6	25.9	40.4 ± 6.4	23	23.5 ± 5.7^$^	24
Jumps (frequency)	8.8 ± 3.4^#ψ^	39	12.5 ± 4.0	32	10.9 ± 5.8^#ψ^	53	11 ± 3.5	32	15 ± 4.7	31
Explosive efforts (frequency)	97 ± 13.8	14	96.4 ± 1.5	1.5	93.2 ± 8.0	8.6	79 ± 14.1^$^	17.7	96.6 ± 1.4^$^	1.4

^$^Significant differences from all other groups; *significant differences from Midfielders; ^#^significant differences from Defenders; ^@^significant differences from Strikers; ^ψ^significant differences from Forwards; ^β^significant differences from Wingers. *p* < 0.05 for all comparisons.

Total distance had differences (*F* = 14.42, *p* ≤ 0.001, ES = 0.63, i.e., large ES); the *S* and *D* groups had a shorter total length than all groups, and *F* had a shorter total length than *M* and *W* groups (*p* ≤ 0.001 for all comparisons).

Statistically significant effects were observed between the positions of players in the distance/min (*F* = 15.06, *p* ≤ 0.001, ES = 0.64, i.e., large ES), where *M* and *F* had higher values than all other groups (*p* ≤ 0.001 for all comparisons).

The total load also showed differences (*F* = 22.39, *p* ≤ 0.001, ES = 0.73, i.e., large ES); the *S* and *D* groups had a lower total load than all the other groups, and *F* had a lower total load than *M* (*p* ≤ 0.001 for all comparisons).

The comparisons also demonstrated effects in load/min (*F* = 19.59, *p* ≤ 0.001, ES = 0.70, i.e., large ES). The *S* and *D* groups had a lower load ratio than all the other groups (*p* ≤ 0.001 for all comparisons).

**FIGURE 1:**
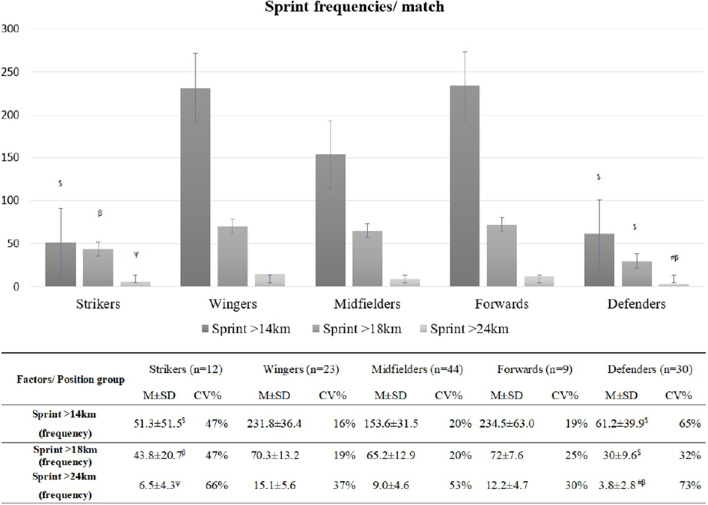
Sprint frequencies by player position. ^$^Significant differences from all other groups; ^#^significant differences from Defenders; ^ψ^significant differences from Forwards; ^β^significant differences from Wingers, *p* < 0.05 for all comparisons.

Figure 1 shows the sprint frequencies per soccer match.

Effects were also observed in sprint frequencies above 14 km/h (*F* = 10.28, *p* ≤ 0.001, ES = 0.55, i.e., large ES), where *S* and *D* had lower frequencies than all other groups (*p* ≤ 0.001 for all comparisons), and *S* had higher frequencies than *D* (*p* = 0.015).

Moreover, the analysis presented differences in sprint frequencies above 18 km/h between the positions of players (*F* = 17.65, *p* ≤ 0.001, ES = 0.64, i.e., large ES), where *S* and *D* had lower frequencies than all other groups (*p* ≤ 0.001 for all comparisons), and *S* had higher frequencies than *D* (*p* = 0.015).

Effects were also observed in sprint frequencies above 24 km/h (*F* = 7.72, *p* ≤ 0.001, ES = 0.48, i.e., large ES), where *D* had lower frequencies than all groups, while *M* had lower frequencies than *S* and *W* (*p* ≤ 0.001 for all comparisons).

Furthermore, the analysis verified differences in maximal velocity comparisons (*F* = 2.41, *p* = 0.007, ES = 0.23, i.e., large ES), *D* had lower speed than *W* (*p* ≤ 0.001).

The comparison also showed differences in the deceleration of sprints (*F* = 7.28, *p* ≤ 0.001, ES = 0.46, i.e., large ES), where *D* had a lower frequency than all other groups (*p* ≤ 0.001 for all comparisons).

Additionally, the analysis observed effects in the acceleration of sprints between the positions of players (*F* = 3.79, *p* ≤ 0.001, ES = 0.31, i.e., large ES), where *D* had a lower frequency than *S* (*p* = 0.005), *W* (*p* ≤ 0.001), *M* (*p* = 0.009), and *F* (*p* = 0.003), and *M* had a lower frequency of acceleration than *W* (*p* = 0.012).

Besides, effects were observed in jump frequencies when comparing the positions of players (*F* = 2.46, *p* = 0.006, ES = 0.23, i.e., large ES), where *S* and *M* had lower frequency than *D* (*p* = 0.003 and *p* = 0.004, respectively).

Finally, the comparisons indicated differences in explosive effort frequencies (*F* = 36.43, *p* ≤ 0.001, ES = 0.56, i.e., large ES); *D* had a lower frequency than other groups (*p* ≤ 0.001 for all), while *M* had lower values than *F* (*p* = 0.032).

**FIGURE 2:**
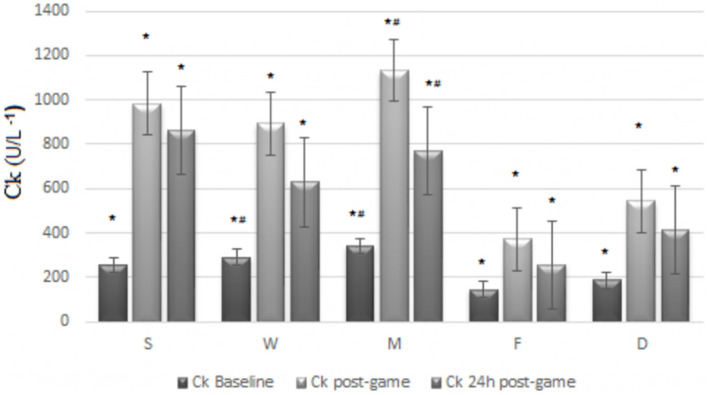
Ck (U/L^–1^) baseline, post and 24 h post game, by player’s position. *Significant differences from all other moments; ^#^significant differences from Defenders; *p* < 0.05 for all comparisons.

Figure 2 shows Ck values. Significant differences were observed between time points when comparing Ck (*X*^2^ = 114.67, *p* ≤ 0.001, ES = 0.59, i.e., large ES). The Ck baseline (255 U/L) time point had lower values than postgame (718.5 U/L, *p* ≤ 0.001) and 24 h postgame (560.0 U/L, *p* ≤ 0.001). Postgame presented higher Ck values than the other time points (*p* ≤ 0.001 for all comparisons). The positions of players demonstrated differences when comparing Ck baselines (*X*^2^ = 30.56, *p* ≤ 0.001, ES = 0.37, i.e., large ES), where *D* (158.0 U/L) had lower values than *W* (341.0 U/L, *p* = 0.003) and *M* (255.0 U/L, *p* ≤ 0.001). Significant differences were observed in Ck postgame (*X*^2^ = 19.89, *p* ≤ 0.001, ES = 0.218, i.e., large ES) and in Ck 24 h postgame (*X*^2^ = 20.55, *p* ≤ 0.001, ES = 0.223, i.e., large ES), where *D* (522.5 and 325.0 U/L) had lower values than *M* (718.5 and 560.0 U/L, *p* ≤ 0.001 for all comparisons) in both Ck postgame and 24 h postgame, respectively.

## Discussion

This study aimed to determine the impact of a soccer game on the Ck response, recovery, and specific running performance outcomes during professional soccer games by comparing playing positions. The main results demonstrated that Ck concentrations were higher at all postgame time points when compared with pregame, with the highest concentrations being observed after the game. Incomplete recovery markers were also identified up to 24 h after the game, especially for midfielders. Significant effects were observed between the positions of players when comparing performance indicators, in which offensive midfielders had higher total and relative distances covered and higher loads during high-level soccer games. The strikers had a lower percentage of submaximum, maximum, and up to maximum limit efforts during the game than other groups. At the same time, middle athletes demonstrated a higher frequency of sprints above 24 km/h, the number of jumps (<30 cm), and the total frequency of explosive efforts. The interactions between the positions and the level and location of opponents were observed for the total distance, relative distance, total load, sprint frequencies above 18 km/h, and decelerations, with higher values in international competitions in South America than at the state level in Rio de Janei ro.

This study showed that midfielders and forwards covered higher distances than other playing positions. This finding is in line with previous studies, which reported a greater distance covered by midfielders, followed by forwards and defenders during a soccer match play (Mohr et al., 2003; Di Salvo et al., 2010; Djaoui et al., 2013; Vescovi and Favero, 2014). The same data were reported in some investigations assessing the French First League, the Spanish La Liga, and the English FA Premier League (Dellal et al., 2010, 2011). For example, Dellal et al. (2010, 2011) investigated the physical activities of elite soccer players across six playing positions. The authors showed that the covered total distances were greater in midfielders (i.e., central defensive midfielders, wide midfielders, and central attacking midfielders) than forwards, central defenders, and full-backs. Furthermore, when analyzing running performance during German *Bundesliga* over three seasons (i.e., 2014/2015, 2015/2016, and 2016/2017) according to five positional roles, Chmura et al. (2018) reported that forwards covered the longer distance in won matches than in drawn and lost matches, while wide midfielders similarly ran a significantly longer distance in drawn and won matches than in lost matches. This finding is also confirmed by Andrzejewski et al. (2019) in their study of 1,178 soccer players taking part in the Polish Premier League matches during the four seasons (from 2010 to 2014). Other data that may support this finding reported that elite midfielders have the biggest intermittent endurance capacity and the maximum rate of oxygen consumption (VO_2__max_) than forwards and defenders (Slimani and Nikolaidis, 2019). This could be explained by the fact that midfielders played in an important position that linked defenders and attackers, which requires them to perform a repetitive moving back and forth between the attack and defense. Practitioners would adopt appropriate specific training plans that adequately elicit heightened cardiovascular demands in midfielders compared with other playing positions.

This study reported that midfielders had a higher sprint frequency and absolute distance sprinting than defenders and attackers. Accordingly, Di Salvo et al. (2010) analyzed 67 European matches (European Champions League and UEFA Cup) over four seasons and compared running performance among five playing positions. The authors found that wide midfielders performed a higher total number of sprints and total sprint distance than other playing positions. In contrast, other studies have shown that wide defenders and attackers covered a significantly greater distance and sprint time than midfielders (Mohr et al., 2003; Rampinini et al., 2008). Other studies by Dellal et al. (2010, 2011) reported that forwards sprinted the greatest distance than other playing positions during the French First League, the Spanish LaLiga, and the English FA Premier League soccer matches. These contradictions may be explained by the fact that each team has a specific playing formation, opposition level, tactics, and physical fitness of players (Al’Hazzaa et al., 2001; Aquino et al., 2017; Sarmento et al., 2018; Slimani et al., 2019; Arjol-Serrano et al., 2021). Therefore, it seems that practitioners would adopt position-specific training programs for their players.

Regarding the acceleration and deceleration according to playing positions, our study found that left/right defenders had lower acceleration and deceleration frequencies than the left/right midfielders, wingers, and strikers. These data confirm the data collected by previous authors (Vigne et al., 2010), who analyzed the activity profiles of players of a top-class team in the Italian National Football League over the course of a season and reported that central defenders perform lower accelerations and decelerations than other playing positions. Another study (Oliva-Lozano et al., 2020) conducted a longitudinal study over 13 competitive microcycles recruiting professional footballers from LaLiga and detecting positional differences in terms of sprint, acceleration, and deceleration profiles. More specifically, greater start speeds than high-intensity accelerations were found in wide midfielders while no statistically significant differences could be reported in central defenders, full-backs, and midfielders. The high-intensity decelerations were performed by midfielders, forwards, full-backs, wide midfielders, and central defenders. Therefore, it seems that practitioners would adopt position-specific training programs that elicit higher acceleration/deceleration in outfielders.

Muscle damage markers, notably Ck, were higher in midfielders compared with defenders immediately and 24 h after the soccer match. Similar results have been reported in the existing scholarly literature with higher Ck immediately after the soccer match in midfielders than other playing positions (Souglis et al., 2018). These data could be explained by the fact that midfielders performed higher acceleration, deceleration, and explosive action than defenders. In contrast, another study (Scott et al., 2016) failed to stratify the Ck levels according to playing positions from 15 elite male soccer players competing in the English Premier League, 48 h following a competitive match. However, based on our findings, practitioners would adopt a position-specific recovery program after the soccer match to return to play as fast as possible.

## Limitations and Strengths

Few studies have investigated Ck profiles in national team players (Hecksteden and Meyer, 2020; Schuth et al., 2021), generally adults (Hecksteden and Meyer, 2020) and more rarely adolescents (Schuth et al., 2021). The present investigation significantly adds to this literature.

However, despite this strength, the sample size is the main limitation of the study since the Strikers and Forwards groups are composed only of three and two individuals. Therefore, individual differences that may modify the outcome distributions of these groups more than the actual differences between groups could influence the ESs reported.

This study presents a further limitation that GPS/LPS substantially underestimated ∼4% of the criterion distance when striding and sprinting over short distances (10 m) at both 1 and 5 Hz (Jennings et al., 2010a,b). In contrast, we were able to control the interactions between the positions and the level and location of opponents. The interactions between the level and location of opponents and the positions were observed in total distance, load, and minutes of the game: in this study, international games presented more, i.e., ∼10% of total load and ∼900 m of total distance than the level of state games. This information could improve the periodization of players associated with international championships. However, despite this, these variables were not the determinant for Ck concentrations. Other limitations include the use of Ck as the only biomarker of muscle damage, while a wider array of biological parameters could have been explored. Further high-quality studies are needed to overcome these limitations.

## Conclusion

Significant effects were observed in terms of the positions of the player when comparing performance indicators, as offensive midfielders had a higher total and relative distance and load during the high-level soccer games. The strikers had a lower percentage of submaximum, maximum, and up to maximum limit efforts during the game than other groups, while defenders demonstrated a higher frequency of sprints above 24 km/h. The forwards showed a higher number of jumps (<30 cm) and a total frequency of explosive efforts. Muscle damage (as assessed by means of Ck levels) did not differ in terms of playing position, suggesting a relevant muscle involvement for every player regardless of his position, up to 24 h after the match. More specifically, according to these findings, no training game format alone is able to develop the overall soccer fitness, with each format eliciting a unique physical load. These results make it possible to create a specific training game according to playing positions, associated with the predominant activities performed during competition. Consequently, practitioners would adopt a position-specific recovery program after the soccer match, particularly for midfielders who are exposed to higher muscle damage after the soccer match play.

## Data Availability Statement

The original contributions presented in the study are included in the article/supplementary material, further inquiries can be directed to the corresponding author/s.

## Ethics Statement

The studies involving human participants were reviewed and approved by 13846919.8.000.5257/Hospital Universitário Clementino Fraga Filho-UFRJ. The patients/participants provided their written informed consent to participate in this study.

## Author Contributions

LF, NE, and DG conceived the study, planned, carried out, and wrote the manuscript. MT, MS, HZ, NB, and BM performed the statistical analysis and reviewed the manuscript. MB realized the manuscript review and formatting. All authors contributed to the article and approved the submitted version.

## Conflict of Interest

The authors declare that the research was conducted in the absence of any commercial or financial relationships that could be construed as a potential conflict of interest.

## Publisher’s Note

All claims expressed in this article are solely those of the authors and do not necessarily represent those of their affiliated organizations, or those of the publisher, the editors and the reviewers. Any product that may be evaluated in this article, or claim that may be made by its manufacturer, is not guaranteed or endorsed by the publisher.
